# Causal links between mitochondrial genes, cerebrospinal fluid metabolites, and delirium: a mendelian randomization study

**DOI:** 10.1055/s-0045-1812892

**Published:** 2025-12-02

**Authors:** Yafeng Wang, Jiaming Wu, Shiyang Wei, Yanyan Hu, Yalan Li

**Affiliations:** 1 Guangxi Academy of Medical Sciences, The People's Hospital of Guangxi Zhuang Autonomous Region, Department of Anesthesiology, Nanning, Guangxi, People's Republic of China.; 2 The First Affiliated Hospital of Jinan University, Department of Anesthesiology, Guangzhou, Guangdong, People's Republic of China.; 3 The First Affiliated Hospital of Jinan University, Department of Neurosurgery, Guangzhou, Guangdong, People's Republic of China.; 4 Guangxi Academy of Medical Sciences, The People's Hospital of Guangxi Zhuang Autonomous Region, Department of Gynecology, Nanning, Guangxi, People's Republic of China.

**Keywords:** Delirium, Cerebrospinal Fluid, Mitochondrial, Metabolite, Mendelian Randomization

## Abstract

**Background:**

Mitochondrial dysfunction plays a crucial role in neuropsychiatric disorders, including delirium.

**Objective:**

To explore the causal links between mitochondrial-related druggable genes, cerebrospinal fluid metabolites, and delirium.

**Methods:**

Summary-level data on mitochondrial-related druggable genes, expression quantitative trait loci (eQTLs), 338 cerebrospinal fluid (CSF) metabolites, and delirium data were obtained from publicly accessible genome-wide association studies. A two-sample Mendelian randomization (MR) was applied to assess the causal effects of blood cis-eQTL of mitochondrial-related druggable genes on delirium. Sensitivity analyses were also undertaken to ensure the MR results' reliability. We assessed whether cerebrospinal fluid metabolites mediate the causal relationship between druggable mitochondrial genes and delirium.

**Results:**

A total of 12 mitochondrial-related druggable genes (8 protective and 4 risk) were identified to be associated with delirium risk ( *p* < 0.05). Furthermore, 20 CSF metabolites were significantly associated with delirium, 9 positively and 11 negatively. Sensitivity analyses showed no evidence of heterogeneity or horizontal pleiotropy. Mediation analysis indicated that 3-hydroxyoctanoate partially mediated the causal association between sterol carrier protein 2 (SCP2) and delirium, accounting for approximately 19.23% of the total effect.

**Conclusion:**

The present work reveals that mitochondrial-related genes and CSF metabolites may play causal roles in delirium and highlights SCP2–3-hydroxyoctanoate as a novel molecular axis. These findings expand current knowledge of delirium pathogenesis and offer a potential molecular target for diagnosis and therapy. Further experimental validation and population-diverse studies are needed to confirm these findings.

## INTRODUCTION

Delirium, characterized by acute confusion and cognitive dysfunction, frequently affects elderly patients with underlying medical conditions. 1 2 While previous research suggests that certain plasma metabolites may be linked to neuropsychiatric disorders, the unique role of cerebrospinal fluid (CSF) metabolites in delirium is still underexplored. This area of study highlights the complex relationship between metabolic disturbances and neuropsychiatric conditions. 3

Recent studies suggest that changes in metabolites could serve as biomarkers for delirium and other cognitive impairments and highlight the therapeutic potential of targeting mitochondrial pathways for therapeutic purposes. 4 5 6 Mitochondria are crucial to cellular energy production and metabolic processes. Their dysfunction is increasingly acknowledged as a key factor in neuropsychiatric disorders, such as delirium. 7 8 Furthermore, research highlights the therapeutic potential of targeting mitochondrial function in neurodegenerative and neuropsychiatric diseases. 9 10

In this context, a two-step, two-sample Mendelian randomization (MR) study can provide insights into the causal relationships between mitochondrial-related therapeutic targets, CSF metabolites, and the incidence of delirium. Investigating these associations through MR can help elucidate whether changes in CSF metabolites causally affect the risk of developing delirium, thereby identifying potential biomarkers for early intervention. Moreover, the interplay between genes, CSF metabolites, and neuropsychiatric disorders has gained attention, with evidence suggesting that metabolites in CSF may influence brain health. A recent study identified a specific CSF marker linked to an increased risk of delirium, suggesting an underlying mechanism. 11

Nonetheless, how these CSF metabolic profiles interact with mitochondrial gene expression in the etiology of delirium requires further elucidation. Understanding the complex relationship between mitochondrial-related druggable genes and neuropsychiatric disorders is an active area of research with implications for both basic biology and the development of novel therapeutics. 12 13 14

Recent years have seen increased interest in investigating mitochondrial-related druggable signatures' roles in diseases like schizophrenia and bipolar disorder. 15 Mitochondrial-related druggable genes encode proteins that can be targeted by therapeutic agents. Investigating how these signatures are connected to delirium can enhance our understanding of the causes and help in developing targeted therapies and personalized care.

Two-sample MR is a modern epidemiological approach that applies genetic variants as instrumental variables to determine potential causal interactions between exposure and outcome variables. 16 By leveraging large-scale genome-wide association studies (GWAS) data and blood cis-expression quantitative trait loci (eQTL) instruments, MR can help disentangle correlation from causation.

In the current study, we performed a two-step, two-sample MR analysis to investigate the association of mitochondrial-related druggable genes with delirium, and the association of CSF metabolites with delirium. Then, we conducted mediation analysis to test whether they serve as intermediaries linking specific mitochondrial-related genes to delirium risk. Our aim is to identify novel causal pathways and potential therapeutic or diagnostic targets for this condition.

## METHODS

Summary-level data from quantitative trait loci and genome-wide association studies are publicly accessible. The research was approved by the institutional review boards of their respective institutions. Figure 1 illustrates the complete design strategy of this study, with detailed explanations of each pathway.

**Notes:Figure 1:**
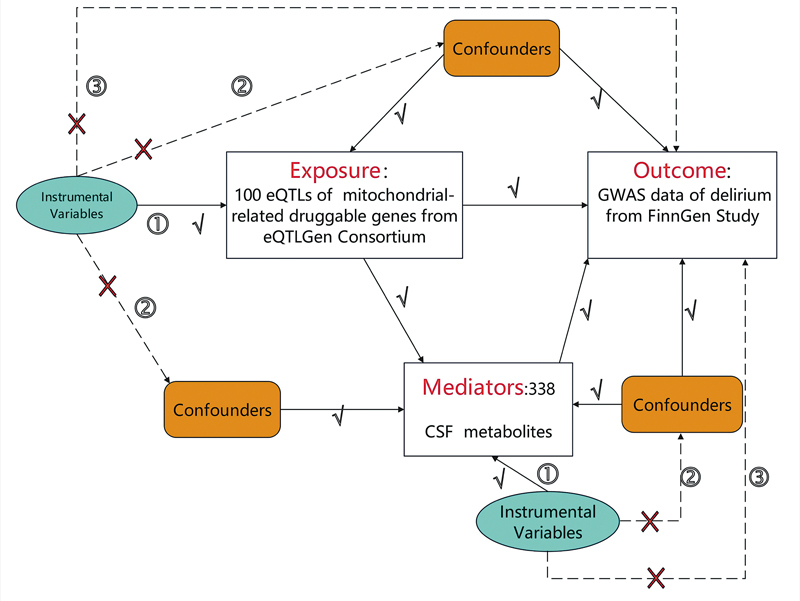
The numbered arrows with checkmarks (√) indicate valid causal pathways: ① represents the association between instrumental variables (IVs) and exposure factors, which must satisfy the relevance assumption (F-statistic >20); ② represents the causal relationship between exposure and outcome through the mediator pathway. The crossed arrows (×) indicate pathways that violate MR assumptions, in ③ representing direct effects of instrumental variables on outcome (violating exclusion restriction), and in ② when appearing with ×, representing eliminated confounding pathways due to random allocation of genetic variants at conception. The dashed box encompasses the overall analytical framework of the two-sample MR approach with mediation analysis. Overview of the study flowchart. This figure illustrates the two-step, two-sample Mendelian randomization (MR) design with mediation analysis framework. Elliptical shapes represent instrumental variables (genetic variants used as proxies). Rectangular boxes represent the main study variables: exposure factors (100 mitochondrial-related druggable genes from eQTLGen Consortium), mediator variables (338 CSF metabolites), and outcome (delirium GWAS data from FinnGen Study). Rounded rectangles represent confounding factors.

### Study design

The MR study design relies on three main assumptions. First, that instrumental variables (IVs) are closely linked to the exposure factors; that they are independent of confounding variables; and that they affect the outcome only through the exposure, without any direct effect.

Based on these three core assumptions, we defined a causal relationship between exposure and outcome. A two-step, two-sample design was used ( Figure 1 ): first, analyzing the effects of mitochondrial-related genes on delirium, then analyzing the effects of CSF metabolites on delirium, followed by mediation analysis of any significant metabolite in the gene–delirium pathway.

### Data sources

#### *Mitochondrial-related druggable genes*

From the MitoCarta3.0 database (Broad Institute), we retrieved 1,136 human mitochondrial-related genes, 17 which were intersected with the druggable gene set of Finan et al., 18 yielding 2,525 genes with cis-eQTL data in blood from the eQTLGen Consortium. 19 We set a minor allele frequency (MAF) > 0.01, and included only variants within ± 1,000 kb of each gene with significant cis-eQTL effects ( *p* < 5 × 10 − 8), as potential instrumental variables.

#### *Cerebrospinal fluid metabolites*

We used publicly available GWAS summary data on 338 CSF metabolites (ID: ebi-a-GCST90025999 to ebi-a-GCST90026336), derived from 291 European participants with no overlap in cohorts. 20 The original CSF GWAS data has been processed using a combined association test (COMBAT) to address batch effects, and we additionally applied genomic control to single-nucleotide polymorphism (SNP) β values before analysis to further minimize potential batch-related biases.

#### *Delirium*

Summary statistics for delirium were obtained from the eleventh public release of data from the Finnish Genomics (FinnGen) study (FinnGen R11), involving 1,083 delirium cases and 445,828 controls of European ancestry, as shown in Table 1 . 21

**Table 1:** Characteristics of data in this study

Trait	Sample size	Population	Data source (PMID)	Description
cis-eQTL	31,684	European	34475573	cis-eQTL data in blood for 100 mitochondrial-related druggable genes
Blood metabolites	8,299	European		
CSF metabolites	291	European	33437055	ID: ebi-a-GCST90025999–ebi-a-GCST90026336, including 338 metabolites
Delirium	431,880	European/Finnish	36653562	ID: finngen_R11_F5_DELIRIUM, including 3,827 delirium patients and 428,053 controls

Abbreviations: CSF, cerebrospinal fluid; eQTL, expression quantitative trait loci; PMID, PubMed.

### Statistical analysis

#### *Two-sample MR analysis*

The SNPs must meet the following criteria to align with core assumptions:

- a suggestive *p* -value of <5 × 10 ^−8^ ;
- an F-statistic of ≥ 20; and
- a minor allele frequency of > 0.01.

In MR analysis, the F-statistic is essential to evaluate the strength of the IVs' association with the exposure variable and identifying potential bias or weak instrument problems. We calculated R ^2^ using the formula: 2 × (1 − MAF) × MAF × β ^2^ , where MAF stands for minor allele frequency and β denotes the effect size on the exposure. The F-statistic was calculated using the formula: F = R ^2^ ×(N − 2)/(1 − R ^2^ ), where N represents the effective sample size.

To avoid linkage disequilibrium bias, SNPs were chosen based on a distance threshold of 10,000 kb and an r ^2^ lower than 0.1. Five MR methods from the TwoSampleMR R package (MRC Integrative Epidemiology Unit), version 0.5.11, were employed to analyze the eQTL and GWAS data, specifically MR Egger, weighted median, inverse variance weighting (IVW), simple mode, and weighted mode.

The IVW approach yields the most reliable outcomes when every SNP meets the fundamental requirement of being a valid instrumental variable. The weighted median technique can estimate the odds ratio (OR) provided that over 50% of the SNPs are valid instrumental variables. The MR Egger method is recommended when multiple validities exist or over 50% of SNPs breach core assumptions. The additional two methods served as complementary techniques. The Wald ratio was applied for individual SNPs.

To ensure our results were robust, we used MR Egger intercept tests and Cochran's Q statistic for pleiotropy and heterogeneity tests, with all *p* -values exceeding 0.05. A leave-one-out method was used to perform an outlier analysis, and the Steiger test was applied to verify the direction of the results.

#### *Method rationale*

We selected IVW as the primary analysis method because it provides the most statistically efficient estimate when all instrumental variables satisfy the MR assumptions. The weighted median method was used as it can provide consistent estimates even when up to 50% of the weight comes from invalid instruments. The MR-Egger was included to detect and adjust for directional pleiotropy through its intercept term, though it has lower statistical power. The MR Pleiotropy Residual Sum and Outlier (MR-PRESSO) was employed to identify and remove outlier instruments that may violate the assumptions. The simple and weighted mode methods were included as supplementary approaches to assess the robustness of our findings (see **Supplementary Material 1** – **Table S1** , available at https://www.arquivosdeneuropsiquiatria.org/wp-content/uploads/2025/09/ANP-2025.0162-Supplementary-Material-1.docx , for a comprehensive comparison of method strengths and limitations).

#### *Mediation analysis*

The study assessed the causal link between mitochondrial-related druggable genes and delirium to identify with significant beneficial effects. A two-sample analysis was conducted to evaluate the causal link between CSF metabolites and delirium, identifying metabolites with positive outcomes. The MR analysis was conducted using positive mitochondrial-related druggable genes as the exposure and positive CSF metabolites as the outcomes ( *p* < 0.05) prior to obtaining the final positive results.

The mediation analysis involved three steps: first, assessing the impact of a significantly associated mitochondrial-related gene (exposure) on a significant CSF metabolites (mediator; β1); second, evaluating the influence of the identified mediators (nused as the exposure) on delirium (outcome; β2); and third, determining the total effect (β0) of mitochondrial-related gene (exposure) identified in the first step on delirium (outcome).

Based on the rule of products of coefficients, the mediation effect, representing the indirect effect of the exposure on the outcome, is calculated as the product of β1 and β2. The mediation percentage was calculated using the formula: (β1 × β2)/β0. Analyses were conducted using R software (R Foundation for Statistical Computing), version 4.3.0.

#### *Sensitivity analysis*

The intercept term from the MR-Egger regression was utilized to indicate the average pleiotropy of instrumental variables, and to assess probability of horizontal pleiotropy. Additionally, MR-PRESSO was used to analyze horizontal pleiotropy. The focus is on recognizing horizontal multivariate validity and adjusting it by removing outliers. We also evaluated whether MR-PRESSO can detect significant changes in causal effects in MR analysis after outliers are removed. To improve the accuracy and robustness of the genetic instrument, we quantified heterogeneity using Cochran's Q statistic, where *p* > 0.05 indicates no effect.

## RESULTS

### Causal effect of mitochondrial-related druggable genes and delirium

From 100 mitochondrial-related druggable genes with available blood cis-eQTL data, 12 genes showed significant associations with delirium risk ( *p* < 0.05 by IVW). There were eight protective genes ( *DHODH* , *DHRS7B* , *TOP1MT* , *CASP8* , *GFM1* , *CPT1B* , *TK2* , *GPD2* ), and four genes ( *SCP2* , *DMPK* , *GSTK1* , *SIRT3* ) that increased delirium risk, as shown in Figure 2 , with *p* = 0.05 (dotted line); and false discovery rate (FDR) < 0.05 (red asterisks).

**Abbreviations:Figure 2:**
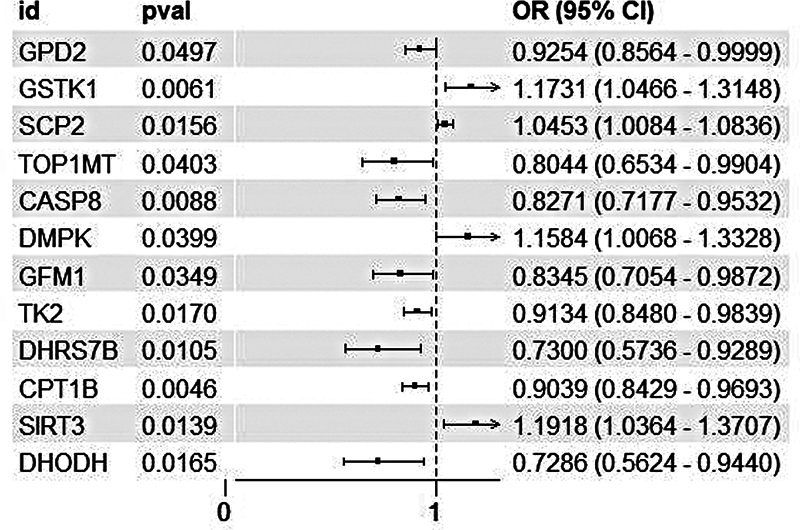
CI, confidence interval; OR, odds ratio. **Notes:** OR = 1, indicates no association between exposure factors and outcomes; OR > 1, indicates that higher gene expression increases the risk of delirium occurrence (risk factors: *SCP2* , *DMPK* , *GSTK1* , *SIRT3* ); OR < 1, indicates that higher gene expression reduces the risk of delirium occurrence (protective factors: *DHODH* , *DHRS7B* , *TOP1MT* , *CASP8* , *GFM1* , *CPT1B* , *TK2* , *GPD2* ). The horizontal lines represent 95% CI for each OR estimate. Forest plot showing the causal associations between 12 significant mitochondrial-related druggable genes and delirium risk estimated by the inverse variance weighted (IVW) method. Results are presented at a significance threshold of *p* < 0.05.

Sensitivity analyses (MR-Egger intercept, Cochran's Q) indicated minimal evidence of horizontal pleiotropy or heterogeneity, except for slight heterogeneity in DMPK (see **Supplementary Material 2** **Table S2** –available at https://www.arquivosdeneuropsiquiatria.org/wp-content/uploads/2025/09/ANP-2025.0162-Supplementary-Material-2.xlsx ).

**Table 2:** Associations between 11 mitochondrial-related druggable genes on 19 CSF metabolites

Exposure	Outcome	nSNP	OR (95%CI)	*p* -value
CASP8	GCST90026066	18	0.921 (0.871–0.974)	0.004
CPT1B	GCST90026174	18	0.968 (0.939–0.997)	0.031
DHRS7B	GCST90026086	6	0.821 (0.697–0.967)	0.018
DMPK	GCST90026203	18	0.929 (0.864–0.999)	0.048
DMPK	GCST90026213	18	1.094 (1.020–1.172)	0.012
DMPK	GCST90026299	18	1.118 (1.022–1.223)	0.015
GFM1	GCST90026203	7	0.832 (0.695–0.996)	0.045
GFM1	GCST90026071	7	0.859 (0.763–0.968)	0.013
GPD2	GCST90026281	32	0.870 (0.762–0.992)	0.037
GPD2	GCST90026086	32	0.919 (0.872–0.968)	0.001
GPD2	GCST90026174	32	0.924 (0.888–0.962)	<0.001
GPD2	GCST90026259	32	0.932 (0.897–0.969)	<0.001
GPD2	GCST90026225	32	0.952 (0.921–0.984)	0.003
GPD2	GCST90026299	32	1.067 (1.000–1.138)	0.049
GPD2	GCST90026044	32	1.092 (1.004–1.189)	0.041
GPD2	GCST90026083	32	1.188 (1.066–1.324)	0.002
GSTK1	GCST90026316	6	1.463 (1.188–1.801)	<0.001
SCP2	GCST90026259	29	0.969 (0.947–0.992)	0.008
SCP2	GCST90026152	29	0.969 (0.950–0.989)	0.003
SCP2	GCST90026214	29	0.970 (0.954–0.987)	<0.001
SCP2	GCST90026120	29	0.974 (0.955–0.994)	0.012
SCP2	GCST90026083	29	1.116 (1.048–1.189)	<0.001
SIRT3	GCST90026316	4	1.437 (1.020–2.025)	0.038
TK2	GCST90026213	17	1.076 (1.008–1.149)	0.029
TK2	GCST90026204	17	1.195 (1.032–1.383)	0.017
TK2	GCST90026316	17	1.218 (1.011–1.467)	0.038
TOP1MT	GCST90026044	12	0.787 (0.640–0.967)	0.023
TOP1MT	GCST90026152	12	0.894 (0.826–0.968)	0.006
TOP1MT	GCST90026207	12	0.915 (0.851–0.983)	0.016
TOP1MT	GCST90026031	12	1.221 (1.074–1.387)	0.002
TOP1MT	GCST90026281	12	1.522 (1.100–2.105)	0.011

Abbreviations: CSF, cerebrospinal fluid; eQTL, expression quantitative trait loci; PMID, PubMed.

### The MR analysis of blood metabolites, CSF metabolites, and delirium

A total of 338 CSF metabolites were tested. Of those, 20 displayed significant causal associations with delirium ( *p* < 0.05), as shown in Figure 3 , with a *p* threshold = 0.05 (dotted line) and FDR < 0.05 (red asterisks). Among these, 9 were positively correlated and 11 were negatively correlated with delirium risk. However, it should be noted that the relatively small sample size ( *n* = 291) for CSF metabolite data may limit statistical power. A post hoc power analysis revealed an average power of 68% to detect moderate effect sizes (OR = 1.5) at α = 0.05, suggesting that some true associations may have been missed. After FDR correction, only these 20 metabolites remained significant, indicating robust associations despite the sample size limitation.

**Abbreviations:Figure 3:**
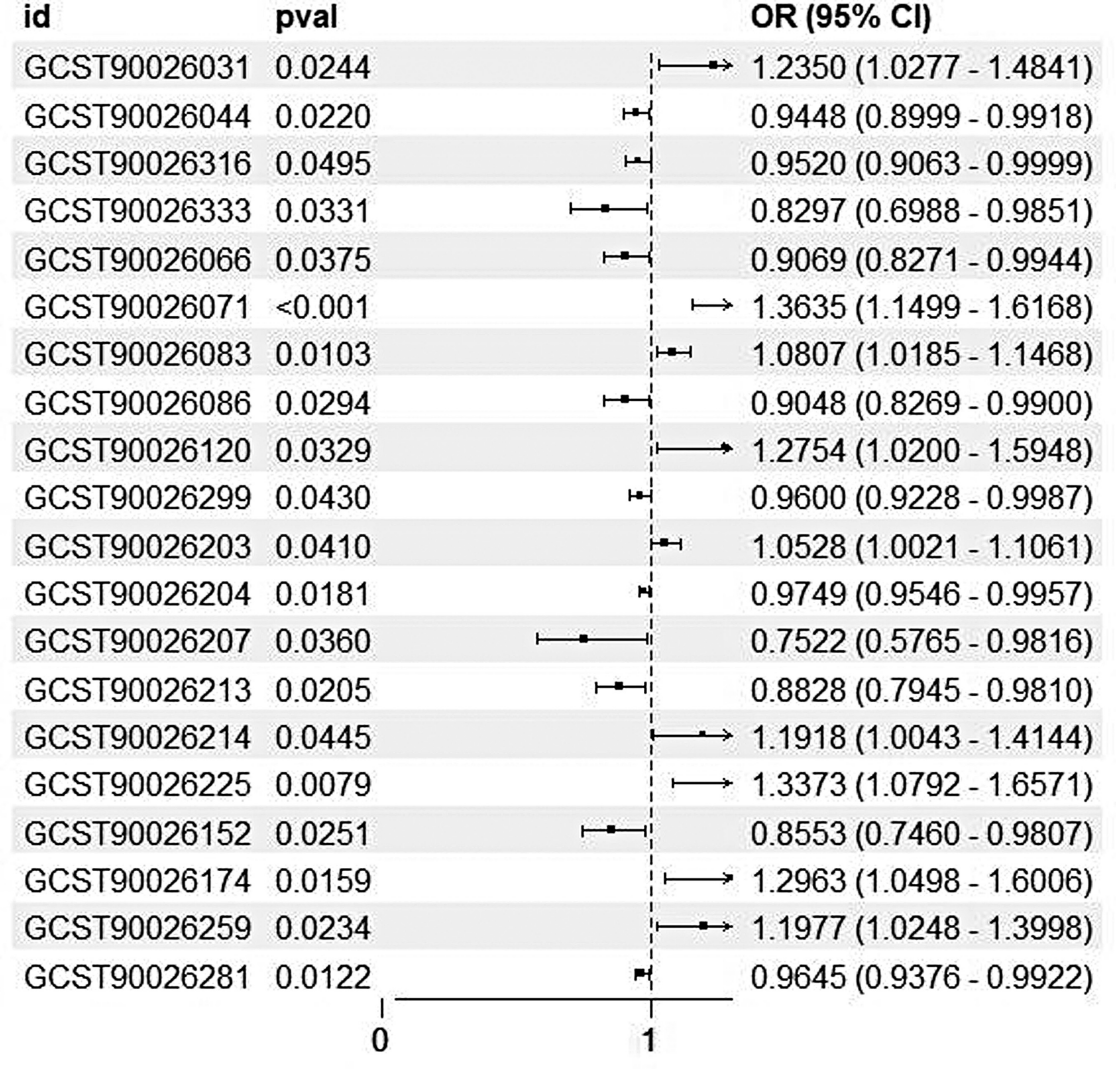
CI, confidence interval; IVW, inverse variance weighted; OR, odds ratio. **Notes:** OR = 1, indicates no association between metabolite levels and delirium; OR > 1, indicates that higher metabolite levels increase the risk of delirium occurrence (risk factors, *n* = 9: GCST90026031, GCST90026071, GCST90026083, GCST90026120, GCST90026174, GCST90026203, GCST90026214, GCST90026225, GCST90026259); OR < 1, higher metabolite levels reduce the risk of delirium occurrence (protective factors, *n* = 11: GCST90026044, GCST90026066, GCST90026086, GCST90026152, GCST90026204, GCST90026207, GCST90026213, GCST90026281, GCST90026299, GCST90026316, GCST90026333). The horizontal lines represent 95% CIs for each OR estimate. Forest plot to estimate the causal association between CSF metabolites and delirium by the IVW method. This plot displays 20 CSF metabolites with significant causal associations with delirium at *p* < 0.05 threshold.

Positively correlated (risk): 2-hydroxy-3-methylvalerate, N-acetylthreonine, glycerophosphoinositol, arabitol/xylitol, hypoxanthine, ribonate, N-acetylglutamine, 3-hydroxyoctanoate, and myo-inositol.

Negatively correlated (protective): N-acetyl-3-methylhistidine, tryptophan betaine, 1-carboxyethylvaline, ethyl β-glucopyranoside, 1-(1-enyl-palmitoyl)-2-arachidonoyl-gpc (p-16 0/20 4), 2'-o-methylcytidine, 3-methoxytyramine sulfate, N-acetylglutamate, dimethylarginine (sdma + adma), X-24813, and N-acetyl-isoputreanine.

Full details are provided in **Supplementary Material 2** **Table S3** .

### Causal relationship between mitochondrial-related druggable genes, circulating metabolites and CSF metabolites

We next examined whether these 12 significant genes are associated with the 20 significant CSF metabolites. There were 11 genes showing causal relationships with 19 metabolites ( *p* < 0.05), as detailed in Table 2 . For instance, sterol carrier protein 2 (SCP2) was positively associated with 3-hydroxyoctanoate but negatively associated with ribonate, asymmetric and symmetric dimethylarginine (sdma + adma), N-acetylglutamine, and arabitol/xylitol.

### Causal link between mitochondrial-related druggable genes and delirium mediated by blood metabolites and CSF metabolites

We identified and measured the CSF metabolites and genes linked to delirium to assess their mediating effects. We calculated the *p* -value for the mediating effects of 20 potential CSF metabolites, as shown in Table 2 . The mediation analysis showed that 3-hydroxyoctanoate levels (GCST90026083) accounted for approximately 19.23% of the impact of SCP2 on delirium risk, with a 95% confidence interval (CI) of 0.49 to 37.97%, and Z = 2.011 ( *p* = 0.044).

The remaining 19 metabolites did not show significant mediation effects, which may be due to: failure to meet all three Baron-Kenny conditions for mediation; inconsistent directional effects between gene-metabolite and metabolite-delirium associations; or insufficient statistical power to detect smaller mediation effects.

These metabolites may influence delirium through alternative pathways not captured in our mediation framework.

## DISCUSSION

This study used Mendelian randomization analysis to investigate the genetic connections between druggable mitochondrial genes, CSF metabolites, and delirium. We found potential causal associations between 12 mitochondrial-related genes, as well as 20 CSF metabolites associated with delirium. We also examined how 12 genes causally affect 20 CSF metabolites.

The results indicated that 11 of these genes were causally associated with 19 CSF metabolites. Additionally, we calculated the proportion of indirect effects using mediation analyses, which suggested that only the level of 3-hydroxyoctanoate had a significant mediating effect of SCP2 on the development of delirium (19.2%, *p* < 0.05). Our research identifies possible mechanisms underlying mitochondrial dysfunction associated with delirium.

The identification of potential mitochondrial-related therapeutic targets provides a new avenue for exploring treatment strategies for delirium. These targets may hold the key to developing more effective interventions aimed at modifying the underlying mechanisms associated with this complex disorder. We analyzed the causal relationship between 100 mitochondrial-related genes and delirium. Our findings show that higher expression of 8 genes, including *DHODH* , *DHRS7B* , *TOP1MT* , *CASP8* , *GFM1* , *CPT1B* , *TK2* , and *GPD2* , could decrease the risk of delirium. Meanwhile, higher expression of *SCP2* , *DMPK* , *GSTK1* , and *SIRT3* might increase the risk of delirium.

Mitochondria are essential for neuronal function, contributing to energy production, calcium regulation, and reactive oxygen species generation. Dysfunction is closely linked to numerous neurological diseases. Mitochondrial-related gene expression offers potential targets for therapeutic intervention. 22 23 Abnormalities are commonly observed in neurodegenerative diseases, including Alzheimer's, 24 Parkinson's, 25 Huntington's, 26 and amyotrophic lateral sclerosis. 27 Understanding and targeting specific druggable mitochondrial genes could lead to the development of innovative therapeutic strategies.

Investigating CSF metabolites can enhance our understanding of the biological processes involved in delirium. We analyzed the causal relationship between 338 CSF metabolites and delirium. The results showed that some increased the risk of delirium, while others provided protective effects. Certain CSF metabolites can serve as important biomarkers for specific neurological disorders, including Parkinson's disease, 28 epileptic spasms, 29 disorders of consciousness, 30 and others.

The relationships observed between specific metabolites and delirium indicate that they could serve as biomarkers or potential therapeutic targets. Alterations in proteins, neurotransmitters, or other molecules can be indicative of diseases such as Alzheimer's, 31 Parkinson's, 32 and multiple sclerosis, 33 aiding in their diagnosis and differentiation.

The levels and alterations of CSF metabolites can provide insights into the underlying pathophysiological processes occurring in the brain. 34 35 They can reflect neuronal damage, 36 inflammation, 37 metabolic disturbances, 38 or neurotransmitter imbalances 39 associated with the disease. It was reported that monitoring changes in CSF metabolites over time can assist in tracking the progression of neurological diseases and evaluating the effectiveness of therapeutic interventions. 40 41

Studying the relationship between CSF metabolites and neurological diseases contributes to a better understanding of the complex mechanisms involved, which can guide the development of new therapeutic strategies. 42 43 Despite these insights, our understanding of them as treatment interventions is still limited. Further research is necessary to clarify the specific mechanisms involved.

Our study revealed a novel SCP2–3-hydroxyoctanoate-delirium molecular axis. The SCP2 is an intracellular lipid transfer protein that facilitates fatty acid transport and mitochondrial β-oxidation. 44 The accumulation of 3-hydroxyoctanoate, a medium-chain fatty acid β-oxidation intermediate, in CSF may reflect impaired mitochondrial function. 45 Given neurons' high dependence on mitochondrial energy production, 22 23 this disruption could contribute to delirium development through compromised synaptic transmission and cellular homeostasis.

The SCP2-mediated dysregulation of lipid metabolism may trigger a cascade of neuroinflammatory responses through the related lipid peroxide-NLRP3-IL-1β pathway, ultimately activating microglial cells. 46 The accumulation of 3-hydroxyoctanoate as a β-oxidation intermediate can induce mitochondrial stress, leading to increased reactive oxygen species production and subsequent activation of the NLRP3 inflammasome. 44 This neuroinflammatory cascade, combined with oxidative stress and disruption of the blood-brain barrier function, creates a pathophysiological environment conducive to delirium. 47

The current study assessed the impact of mitochondrial-related druggable genes on delirium by analyzing their relative expression to determine if they were beneficial or detrimental. While the precise mechanism through which druggable genes related to mitochondria aid in delirium intervention remains to be fully elucidated, our mediation analysis found that 3-hydroxyoctanoate levels accounted for 19.2% of the causal relationship between SCP2 and delirium, demonstrating a mediating effect of 0.009 ( *p* < 0.05). These findings suggest that targeting the SCP2–3-hydroxyoctanoate axis could represent a novel therapeutic strategy for delirium, and CSF β-oxidation intermediates might serve as potential biomarkers.

### Future directions

To validate our findings, we propose several experimental approaches. First, in vitro studies using iPSC-derived neurons with SCP2 overexpression or knockdown to measure 3-hydroxyoctanoate levels, reactive oxygen species production, and mitochondrial membrane potential. Also, in vivo validation using SCP2 knockout mice subjected to LPS-induced acute inflammation to assess behavioral changes and CSF metabolic profiles. Finally, the clustered regularly interspaced short palindromic repeats (CRISPR) screening or small interfering RNA (siRNA) library approaches to systematically validate the causal network between mitochondrial genes, CSF metabolites, and delirium phenotypes.

These experimental validations would provide mechanistic insights beyond the statistical associations identified in our MR analysis.

This study is the first to use a comprehensive MR framework to explore the causal links among mitochondrial-related therapeutic targets, CSF metabolites, and delirium. We conducted a two-step MR analysis followed by mediation analysis. The study used several sensitivity analyses to strengthen the reliability of the MR findings.

The implications of these findings are significant. Future studies can focus on validating these mitochondrial-related genes in independent cohorts and conducting functional experiments to elucidate their precise mechanisms of action. Additionally, exploring the potential of targeting mitochondrial-related genes for therapeutic intervention holds promise for improving patient outcomes.

However, important limitations must be noted.

First, our GWAS data were predominantly from European cohorts, which may limit the generalizability of results to other ethnicities. Population-specific differences in linkage disequilibrium structure and allele frequencies could lead to biased effect estimates when extrapolated to non-European populations. Future studies should leverage multi-ancestry GWAS resources, such as the population architecture through genomics and environment (PAGE) consortium and BioBank Japan for replication analyses. If data availability permits, we plan to conduct MR-MEGA or mixed-ancestry MR sensitivity analyses to assess the consistency of our findings across different populations.

Regarding MR assumptions, although we conducted pleiotropy and heterogeneity tests, residual horizontal pleiotropy might still bias causal estimates. Additionally, unmeasured confounding factors, such as medication history, inflammatory status, and gene-environment interactions, could influence eQTL expression patterns. While MR leverages the random allocation of genetic variants at conception to minimize confounding factors, it cannot account for gene-environment interactions. Future studies should consider multivariable MR or negative control analyses to address these limitations.

This study did not provide direct mechanistic experiments to confirm the biological interaction between SCP2 and 3-hydroxyoctanoate in delirium. Future research should include functional assays and expanded multi-ethnic cohorts.

We used publicly available summary data from multiple studies, so potential batch effects could influence the metabolite's measurements. Although the original CSF GWAS applied COMBAT correction and we performed additional genomic control, residual effects may persist. Future meta-analyses should implement harmonized quality control pipelines and consider meta-regression approaches to systematically address batch-related heterogeneity.

Finally, this study had limited statistical power. The CSF metabolite GWAS sample size of 291 individuals may have resulted in insufficient power to detect associations with smaller effect sizes. Future studies should aim to expand sample sizes through meta-analysis of multiple CSF metabolomics cohorts to improve the reliability and generalizability of findings.

In conclusion, the present study investigated the causal relationships between mitochondrial-related therapeutic targets, CSF metabolites, and delirium. The study identified positive ( *n* = 4) and negative ( *n* = 8) causal effects between genetic liability in mitochondrial-related druggable genes and delirium. There were positive ( *n* = 9) and negative ( *n* = 11) causal effects of CSF metabolites. Additionally, the mediation analysis revealed that CSF 3-hydroxyoctanoate levels mediate the causal effect of SCP2 on delirium. The findings highlight the importance of mitochondrial function and CSF metabolites in the pathogenesis of delirium.
